# Supplementary material for: Modeling glioblastoma heterogeneity as a dynamic network of cell states
Source: Mol Syst Biol. 2021 Sep 16;17(9):e10105. doi: 10.15252/msb.202010105 (PMC8444284; doi:10.15252/msb.202010105)
Supplement: Supplementary file 6 — Source Data for Figure 5 [file MSB-17-e10105-s004.zip › Figure5A_sourcedata/GSEA_3071/hallmarks_stateIV.GseaPreranked.1623742551702/HALLMARK_COAGULATION.html]

Details for gene set HALLMARK\_COAGULATION[GSEA]

|  || Dataset | state53071 |
| Phenotype | NoPhenotypeAvailable |
| Upregulated in class | na\_neg |
| GeneSet | HALLMARK\_COAGULATION |
| Enrichment Score (ES) | -0.5071626 |
| Normalized Enrichment Score (NES) | -2.1519933 |
| Nominal p-value | 0.0 |
| FDR q-value | 1.5357143E-4 |
| FWER p-Value | 0.001 |
Table: GSEA Results Summary

  

Fig 1: Enrichment plot: HALLMARK\_COAGULATION      
 Profile of the Running ES Score & Positions of GeneSet Members on the Rank Ordered List

  

| PROBE | GENE SYMBOL | GENE\_TITLE | RANK IN GENE LIST | RANK METRIC SCORE | RUNNING ES | CORE ENRICHMENT || 1 | CLU |  |  | 27 | 2.064 | 0.0425 | No |
| 2 | PDGFB |  |  | 86 | 1.615 | 0.0687 | No |
| 3 | DUSP6 |  |  | 156 | 1.359 | 0.0869 | No |
| 4 | A2M |  |  | 188 | 1.283 | 0.1106 | No |
| 5 | PLAT |  |  | 281 | 1.070 | 0.1178 | No |
| 6 | CD9 |  |  | 288 | 1.062 | 0.1412 | No |
| 7 | CAPN5 |  |  | 383 | 0.923 | 0.1446 | No |
| 8 | P2RY1 |  |  | 400 | 0.907 | 0.1625 | No |
| 9 | ADAM9 |  |  | 984 | 0.528 | 0.0636 | No |
| 10 | C1R |  |  | 1072 | 0.502 | 0.0586 | No |
| 11 | HTRA1 |  |  | 1445 | 0.404 | -0.0029 | No |
| 12 | FYN |  |  | 1483 | 0.394 | -0.0009 | No |
| 13 | PROS1 |  |  | 1609 | 0.367 | -0.0162 | No |
| 14 | USP11 |  |  | 1665 | 0.356 | -0.0185 | No |
| 15 | CFI |  |  | 2008 | 0.295 | -0.0768 | No |
| 16 | F12 |  |  | 2038 | 0.291 | -0.0756 | No |
| 17 | LGMN |  |  | 2610 | -0.284 | -0.1779 | No |
| 18 | ITGB3 |  |  | 2905 | -0.319 | -0.2265 | No |
| 19 | FURIN |  |  | 2938 | -0.324 | -0.2251 | No |
| 20 | S100A13 |  |  | 3219 | -0.363 | -0.2701 | No |
| 21 | MAFF |  |  | 3620 | -0.432 | -0.3363 | No |
| 22 | PEF1 |  |  | 3940 | -0.496 | -0.3856 | No |
| 23 | WDR1 |  |  | 4413 | -0.641 | -0.4608 | No |
| 24 | BMP1 |  |  | 4563 | -0.713 | -0.4727 | No |
| 25 | F2RL2 |  |  | 4635 | -0.759 | -0.4687 | No |
| 26 | DUSP14 |  |  | 4838 | -0.904 | -0.4863 | Yes |
| 27 | CTSB |  |  | 4948 | -1.012 | -0.4837 | Yes |
| 28 | MMP14 |  |  | 4983 | -1.064 | -0.4657 | Yes |
| 29 | SH2B2 |  |  | 5076 | -1.233 | -0.4547 | Yes |
| 30 | CAPN2 |  |  | 5078 | -1.236 | -0.4264 | Yes |
| 31 | TIMP1 |  |  | 5086 | -1.257 | -0.3987 | Yes |
| 32 | CSRP1 |  |  | 5103 | -1.304 | -0.3717 | Yes |
| 33 | LRP1 |  |  | 5152 | -1.437 | -0.3477 | Yes |
| 34 | SERPINE1 |  |  | 5162 | -1.501 | -0.3148 | Yes |
| 35 | PRSS23 |  |  | 5179 | -1.562 | -0.2818 | Yes |
| 36 | THBS1 |  |  | 5222 | -1.800 | -0.2482 | Yes |
| 37 | F3 |  |  | 5230 | -1.856 | -0.2067 | Yes |
| 38 | ITGA2 |  |  | 5234 | -1.908 | -0.1633 | Yes |
| 39 | CFH |  |  | 5256 | -2.163 | -0.1174 | Yes |
| 40 | FBN1 |  |  | 5263 | -2.334 | -0.0647 | Yes |
| 41 | FN1 |  |  | 5284 | -3.017 | 0.0011 | Yes |
Table: GSEA details [plain text format]

  

Fig 2: HALLMARK\_COAGULATION: Random ES distribution      
 Gene set null distribution of ES for **HALLMARK\_COAGULATION**
